# Thermal Alternating Polymer Nanocomposite (TAPNC) Coating Designed to Prevent Aerodynamic Insect Fouling

**DOI:** 10.1038/srep38459

**Published:** 2016-12-07

**Authors:** Ilker S. Bayer, K. Ghokulla Krishnan, Robert Robison, Eric Loth, Douglas H. Berry, Thomas E. Farrell, Jeffrey D. Crouch

**Affiliations:** 1 Smart Materials, Instituto Italiano di Tecnologia, Genoa, Italy; 2 Mechanical and Aerospace Engineering, University of Virginia, VA, USA; 3 The Boeing Company, Seattle, WA, USA

## Abstract

Insect residue adhesion to moving surfaces such as turbine blades and aircraft not only causes surface contamination problems but also increases drag on these surfaces. Insect fouling during takeoff, climb and landing can result in increased drag and fuel consumption for aircraft with laminar-flow surfaces. Hence, certain topographical and chemical features of non-wettable surfaces need to be designed properly for preventing insect residue accumulation on surfaces. In this work, we developed a superhydrophobic coating that is able to maintain negligible levels of insect residue after 100 high speed (50 m/s) insect impact events produced in a wind tunnel. The coating comprises alternating layers of a hydrophobic, perfluorinated acrylic copolymer and hydrophobic surface functional silicon dioxide nanoparticles that are infused into one another by successive thermal treatments. The design of this coating was achieved as a result of various experiments conducted in the wind tunnel by using a series of superhydrophobic surfaces made by the combination of the same polymer and nanoparticles in the form of nanocomposites with varying surface texture and self-cleaning hydrophobicity properties. Moreover, the coating demonstrated acceptable levels of wear abrasion and substrate adhesion resistance against pencil hardness, dry/wet scribed tape peel adhesion and 17.5 kPa Taber linear abraser tests.

One of the most common examples of insect residue is the car windshield contamination due to insect collisions on the highway^1^. Although this is mostly an annoyance for drivers, avoiding insect residue accumulation due to collisions with moving surfaces has many important technological implications^2^. In aerodynamic architectures such as wings or turbine blades, performance levels are closely related to the airflow boundary layer attached to the airfoil section where turbulent flow has a higher drag than laminar flow^3^. For instance, for wind turbines installed in warm and humid climates, insect collisions with the blades can foul blade surfaces leading to a marked increase in skin drag and reducing power production by as much as 50%^4^. In aircraft flight, surface contamination due to insect impact on wing leading edge surfaces may cause the boundary layer to prematurely transition to turbulent flow, resulting in a potential increase in flow drag and fuel consumption^5^,^6^,^7^,^8^,^9^,^10^. As such, designing new surfaces or coatings for aerodynamic architectures that can resist insect fouling may reduce operational costs^11^,^12^,^13^,^14^.

At high speeds, insect collisions result in debris accumulation in the form of biological body and tissue fragments as well as fluids. Early solutions proposed injection of liquids or surfactants and even anti-icing fluids onto the leading edge^15^,^16^,^17^,^18^,^19^,^20^ but this proved not to be practical. With the recent advances in liquid repellent surfaces, there has been renewed interest in the general problem of self-cleaning and easy cleaning coatings and in particular, surface treatments or coatings that can also withstand insect fouling^21^,^22^. However, outcome of insect impact on surfaces is complicated and cannot be simply associated or modelled with the dynamics of liquid droplet impact and bounce-back over a non-wettable surface as many different types of biological tissues and fluids are involved^23^. Consequently, the use of coatings to mitigate insect residue adhesion requires more attention and detailed investigation. The most general approach includes physicochemical processes to modify a surface to provide reduced adhesion properties to mitigate insect residue adhesion. This can be achieved by topographically and chemically modifying the surface with a low surface energy coating. Whether the treatment is made by physical and chemical modification of metal surfaces or by means of a non-wettable coating, the most important aspect of the coating is the texture and the associated roughness levels^24^,^25^. The most general term for a non-wettable coating is superhydrophobicity. Superhydrophobic coatings generally display static water contact angles greater than 150° and with very low droplet roll-off angles^26^ (upon tiling) preferably below 5°. Coatings that also repel other liquids such as oils and hydrocarbon liquids are known as superoleophobic^27^. As shown in this work a superoleophobic surface or coating may not be absolutely essential for insect impact repellency.

There are a number of recent works which looked at different types of hydrophobic and superhydrophobic surfaces in reducing insect impact residues. The leading work was done at NASA with fruit flies on a variety of low surface energy coatings^28^. NASA researchers evaluated a range of surfaces including commercial siliconized and fluorinated coatings developed for the protection of electronic circuit boards and found that a rougher surface influences the spreading mechanism of the hemolymph, such that a rough low surface energy coating could be used to inhibit the hemolymph from spreading upon impact with the surface. A low surface energy, hydrophobic surface, is imperative as a liquid spreading on a rough surface with high surface energy would essentially spread out (under the Wenzel wetting regime)^29^, whereas a liquid spreading on a rough surface with low energy would resist spreading adopting a Cassie–Baxter wetting state^30^. NASA researchers verified this implication by examining the results obtained using the superhydrophobic surfaces (polymeric and titanium nanotube surfaces)^31^,^32^,^33^. It was found that the superhydrophobic surfaces did not necessarily mitigate insect residue adhesion and that the performance of some superhydrophobic coatings (ranked by residual area measurement) was similar to that of smoother hydrophobic coatings like Teflon and that increased performance tended towards submicron scale surface roughness. Optimization of a surface, both chemically (to obtain a low surface energy) and topographically, is therefore necessary to reduce insect residue adhesion^34^. Consequently, investigation of the optimum topography (i.e. roughness) still remains necessary to ensure design of surfaces or coatings with complete mitigation of insect residue adhesion^35^,^36^ while not being too rough to inherently produce turbulent flow. To achieve this, we created the required surface texture by anchoring superhydrophobic nanoparticle films on a hydrophobic thermoplastic polymer surface. This was accomplished by developing a thermal, alternating polymer nano-composite (TAPNC) coating. The superhydrophobic nanoparticle film is in fact the outermost layer of the TAPNC coating made by subsequent spray painting of alternating layers of polymer and nanoparticle solutions and thermally treating each applied layer afterwards.

## Experimental

### Materials and Methods

Capstone ST-100 was purchased from DuPont; it is an aqueous dispersion of perfluoroalkyl methacrylic copolymer (PMC), containing 74–81% water and 19–26% of PMC. Initial tests revealed that the as-received dispersion cannot be used to create the layer-by-layer coatings due to surfactants used to maintain stable polymer dispersion in water. It was therefore desired to precipitate the PMC and re-disperse it in other solvents in an effort to find a solvent for PMC such that the solution was capable of being spray painted. Therefore, PMC was precipitated from the original aqueous dispersion by mixing equal volumes of Capstone ST-100 and trifluoroacetic acid (Sigma-Aldrich), disrupting colloidal stability. Upon precipitation, the supernatant was decanted and the polymer precipitate (in the form of a rubbery state) was washed several times with water and ethanol and dried in a plastic desiccator^37^. Acetone was found to be a suitable solvent for PMC, and was able to produce uniform coatings. It should be noted that adding other co-solvents with higher boiling points helped in spraying more uniform polymer films. The most suitable solvents were tetrahydrofuran, THF, and methyl ethyl ketone, MEK. Solutions with concentrations from 15 to 30 wt% of PMC in acetone were prepared in increments of 5%. To prepare the polymer/nanoparticle composites or just the nanoparticle films, commercially available hydrophobic fumed silica nanoparticles (Aerosil R812, Evonik) were either dispersed in the PMC/acetone solutions or separately dispersed in chloroform. Polymer/nanoparticle solutions were sonicated in an ultrasonic bath for 30 minutes before spray coating. Polymer, polymer/nanoparticle and nanoparticle solutions were sprayed onto aluminum substrates using an internal mix, double-action airbrush atomizer (model VL-SET, Paasche). The thickness of the polymer or nanoparticle films can be tuned independent from one another by spraying from solutions that are more or less concentrated in polymer and/or nanoparticles, as well as by applying few or more spray passes.

**Figure 1:**
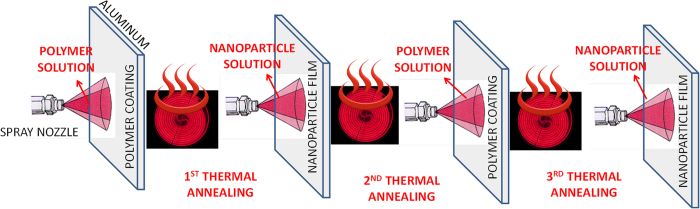
Schematic representation of sequential spray coating process and heating of alternating layers of polymer coating and nanoparticle film. A final thermal annealing is required once the desired layer of alternating coatings is complete (not necessarily three as shown schematically above).

The coatings for insect impact experiments were prepared on aluminum surfaces by spray painting alternating layers of PMC and silica (SiO_2_) nanoparticles as shown in Fig. 1. The thickness of the polymer layer, once dry, was kept around 8–10 μm. After a number of preliminary tests, the optimum final nanoparticle layer thickness was determined to be 300 nm or less. As many as 6 alternating layers can be applied and a minimum of 4 layers performed the best in repelling impacting fruit flies. Between each step thermal treatment (melting) of the thermoplastic polymer was performed to ensure that portion of the nanoparticle film can be embedded into the polymer matrix. Figure 1 schematically describes this process.

**Figure 2:**
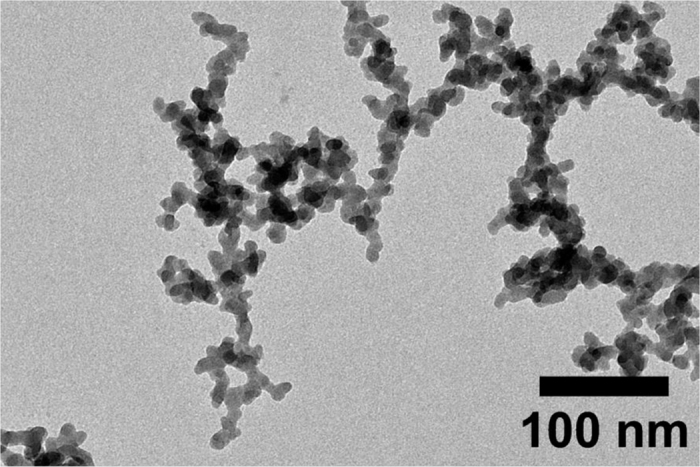
TEM micrograph of as-received Aerosil R812 fumed silicon dioxide nanoparticles.

The morphology of the produced coatings was characterized by two different scanning electron microscopes (SEM): JEOL JSM-6490LA and JEOL 6700F FESEM microscopes working in high-vacuum mode, with an accelerating voltage of 10–20 kV. For scanning electron microscopy measurements, samples were coated with a 12 nm thick layer of Au/Pd to reduce surface charging. Where necessary, samples were tilted at various angles to bring out differences in morphology. Transmission electron microscopy (TEM) was performed with a JEOL JEM-1011 under an accelerating voltage of 100 kV. All the samples for TEM analysis were prepared by immersing carbon-coated 200 mesh, 50 μm copper grids in the nanoparticle dispersions, and then allowing to dry overnight under inert atmosphere. A representative TEM image of the silica nanoparticles is given in Fig. 2.

Atomic force microscopy (AFM) measurements were performed with a Park Systems XE-100 in noncontact mode in order to measure the surface roughness of the coating before installing in the wind tunnel. At least three different zones were chosen on each sample to extract the average roughness values and at least three different samples were measured. Acquired AFM topography images were processed by public domain software known as WS × M developed for data acquisition and processing in scanning probe microscopy.

To measure the contact angle, roll-off angle, and contact angle hysteresis of the coatings, solutions were spray painted on 25 × 50 mm pieces of 25 μm thick aluminum. Five contact angle measurements were taken on random locations of the coatings with 10 μL deionized water droplets using a contact angle goniometer (OCA 20, Dataphysics) equipped with a charge-coupled device (CCD) camera and image processing software. Reported contact angle measurements were averages of five measurements on each sample. Roll-off angle and contact angle hysteresis were measured five times at random locations, and their average, minimum, and maximum values are reported.

Linear abrasion experiments were conducted with a Taber Linear Abraser 5750. The applied weight was 0.35 kg with stroke length of ~5 cm and stroke speed of 15 cycles/min. The abradant used was Calibrase Disk (CS-10F) with a base diameter of approximately 1.5 cm. It is a resilient disk composed of a binder and aluminum oxide abrasive particles that offers a mild abrading action, designed to operate under loads of 0.25 to 0.5 kg, corresponding to roughly 13 kPa to 29 kPa abrasion pressures, respectively. The CS-10F abradant is typically used to test safety glazing materials and transparent plastics against abrasion induced transparency losses. In this work, the corresponding abrasion pressure is 17.5 kPa under 0.35 kg of applied weight.

**Figure 3:**
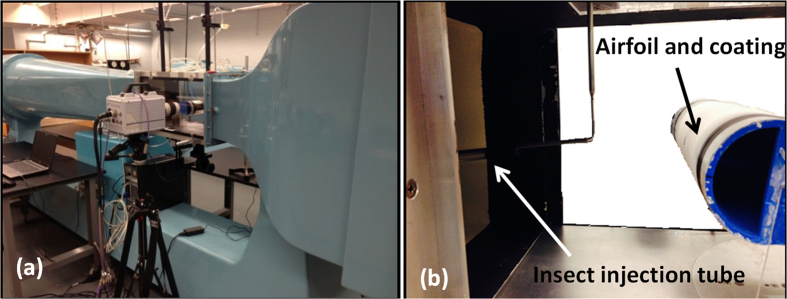
Experimental wind tunnel. (**a**) Photograph of the wind tunnel and the test section with transparent side windows and (**b**) side view close-up of the injection tube in the wind tunnel. On the right the airfoil and the coating are visible.

The experimental setup for insect impact outcome analysis used in this study is shown in Fig. 3 and consists of a wind tunnel, airfoil, high-speed camera (Photron SA4, USA running at 3600 fps or higher when necessary), light source and injection tube. A symmetric modified NACA0038 airfoil with a chord length of 21 cm and leading edge radius (R) of 4.0 cm was used in this study. The wind tunnel cross section was 31 cm × 31 cm.

Based on previous studies, flightless fruit flies of the order Diptera were chosen as a representative insect. The flightless fruit flies were obtained from a local pet store and had an average mass of 0.7 mg. Approximating these fruit flies as ellipsoids, the fruit flies were measured to have axis diameters of d1 = 2.01 mm (length-wise), d2 = 0.92 mm (span-wise) and d3 = 0.85 mm (height-wise). This gives an equivalent volumetric diameter (d) of 1.16 mm. The density of the fruit fly was calculated to be 850 kg/m^3^. The fruit flies were fed upstream of the wind tunnel inlet into a clear Tygon^®^ tube with outer diameter of 9.5 mm. Compressed air was employed using a Venturi nozzle so that the insects reached a velocity of 47 m/s in the test section (obtained from high-speed video analysis), a speed which corresponded to the test section air flow speed. The injection tube exit was placed 18.5 cm away (~90 body lengths) from the stagnation point of the airfoil. This distance minimized the wake effects arising from the injection tube itself. The insects were released in batches of 5 at a time until a total insect release of 50 (i.e. 10 batches of 5 fruit flies) or until a congested strike zone was observed on the coating. A congested strike zone is defined as enough visible insect residues on the coating where an additional impact may have a significant likelihood of occurring on top of a pre-existing residue instead of the coating. This was done so that all impacts occurred on the uncontaminated portion of the coating as opposed to a pre-existing residue since the goal of the study was to quantify the effectiveness of the coating. At least two similarly prepared replicates (two sheets cut from a larger piece) of each coating were tested in the wind tunnel for consistency in the results. After the first 50 insects were released, the coated surface was removed from the wind tunnel and optically analyzed. Afterwards another set of 50 insects were impacted on an identical surface in the wind tunnel. In total 100 insect impact events were allowed on each surface. The insect residues accumulated on the surfaces were characterized using a Hirox KH-7700 digital microscope.

### Screening experiments prior to the development of the TAPNC coating

**Figure 4:**
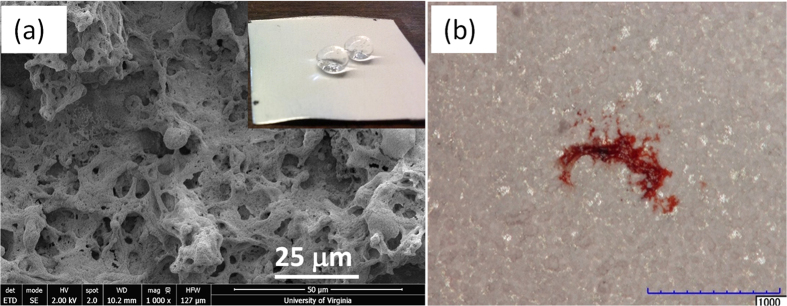
(**a**) SEM image of PMC/silica nanocomposite coating with ~25% nanoparticle concentration (dry basis). The inset is a photograph of water drops on the coating. (**b**) Optical microscope image of a fruit fly residue on the same surface. The scale bar is 1 mm.

The present TAPNC coating was designed as a result of performance experiments conducted on various types of PMC-silica nanocomposites with varying polymer-to-nanoparticle filler ratios. In our earlier works, we have prepared superhydrophobic and oleophobic polymer nanocomposites by blending various fumed silica nanoparticles with PMC used in the present study as the intermediate layer. Although, these coatings performed very well in repelling water and a large number of other liquids including oils and edible sauces and juices, they featured micro-textured (tens of microns) surface topography. They displayed wear abrasion resistance performance similar to that reported by Milionis *et al*.^38^ but did not perform well in the wind tunnel tests with significant amount of insect residue accumulation. In order to reduce the roughness features to sub-micro textured (~1 micron) levels, co-solvents were introduced in order to control the evaporation of the spray mist droplets. As such, the “wetness” of the spray deposited film or composites could be adjusted. In general, polymer nanocomposites that are deposited from rapidly evaporating solvents such as acetone or chloroform result in formation of very dry and flaky coating structures. Wet coatings on the other hand allow a certain degree of self-assembly that can favor lower surface roughness values or much narrow surface roughness distribution^39^. However, an unacceptable level of insect residue accumulation was still detected as seen in Fig. 4. Oil infused micro-textured surfaces fabricated according to reported recipes in the literature was also tested but many exoskeleton attachments were observed on these surfaces^40^.

## Results and Discussion

**Figure 5:**
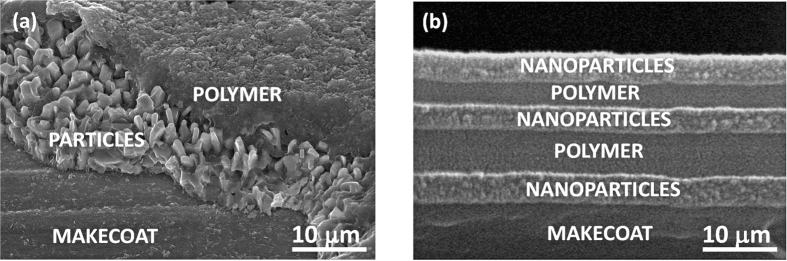
Examples of sequential polymer-micro/nanoparticle film layers forming a composite coating. (**a**) SEM image of multilayer polymer-micro-particle coating and (**b**) SEM image of a multilayer polymer nanoparticle film coating.

In order to render the nanoparticle coating stable against mechanical abrasion, the durability of the makecoat (the first polymeric layer) is very important. It can be a thermoplastic polymer or a heat activated adhesive. The PMC polymer used in this study displayed can be melted and cooled repeatedly as well as demonstrating good adhesion to the substrate. The thermal characteristics of the polymer is presented in a previous work in which electrospun fibers of PMC were melted in order to form oleophobic coatings on aluminum^38^,^41^. The adhesion strength of the polymer to steel was previously reported to be approximately 1750 N/m based on 90° tape peel tests^42^. The nanoparticle film must be applied while the makecoat is melted or in a semi-solid state so that a layer of nanoparticles can be embedded into this polymer layer. Figure 5 shows SEM images of multilayer coatings fabricated both by micron sized particles (Fig. 5a) and nanoparticles (Fig. 5b). As mentioned earlier, the thickness of the nanoparticle film can be adjusted by either changing the concertation of the nanoparticles in the spray solution or by decreasing the number of spray passes over the surface during coating application. Spray application and the solution concentration were adjusted such that coatings made by six or more alternating layers featured a total thickness of 30–50 μm. In the example of Fig. 5b, the coating has a net thickness of 30 μm.

**Figure 6:**
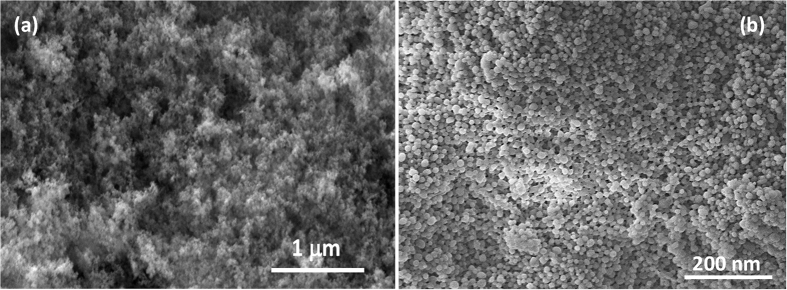
SEM images of nanoparticle film surface morphology. (**a**) As sprayed micron scale surface topography of the outermost layer of a multilayer coating and (**b**) micron scale surface topography of the outermost layer of a multilayer coating after thermal annealing that is able to minimize and mitigate insect residue accumulation.

Figure 6a demonstrates the surface morphology of the outmost layer of a hydrophobic silica layer just after it was spray deposited and before thermal treatment. If no thermal treatment is used, this nanoparticle film could be easily removed from the surface. Upon thermal treatment and removal of the non-stick portion of the nanoparticles by pressure dust gun, the morphology of the nanoparticle film changes drastically as shown in Fig. 6b. After the final thermal treatment just before installing in the wind tunnel for insect impact experiments, the average surface roughness of the superhydrophobic coatings was determined to be around 500 nm by AFM measurements.

**Figure 7:**
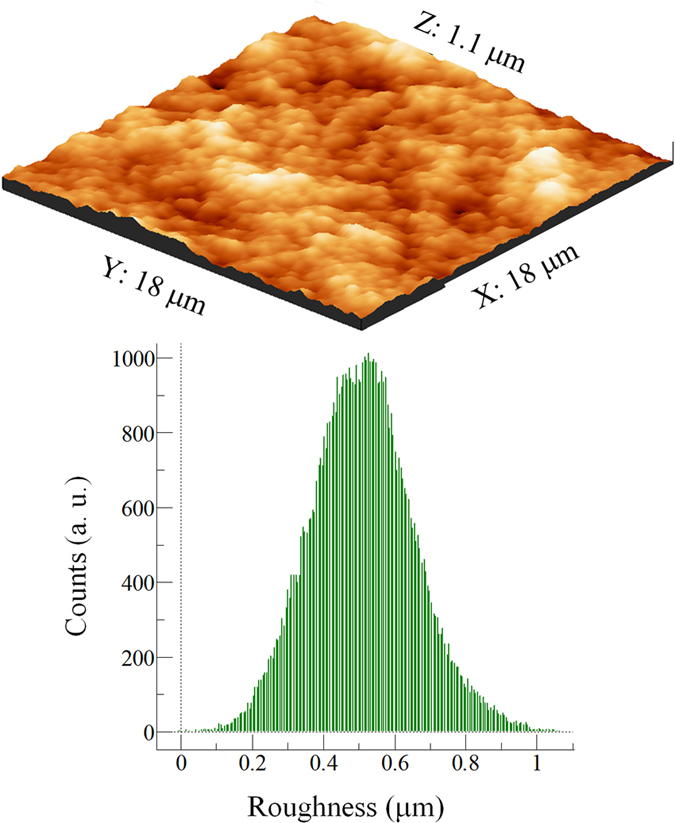
Top: Representative 3D AFM topography image of the superhydrophobic coating before testing (without insect residue). The bottom graph represents the roughness histogram obtained from the topography image above. Corresponding median roughness of ~500 nm was determined.

Figure 7 demonstrates the surface topography of the superhydrophobic coatings that were used for insect impact experiments in the wind tunnel.

Both the topography feature and the average roughness values are displayed. Based on several AFM measurements on different surfaces produced by the same fabrication process, the average surface roughness was determined to be approximately 500 nm.

### Durability screening against abrasion wear, adhesion and water soaking. 

**Figure 8:**
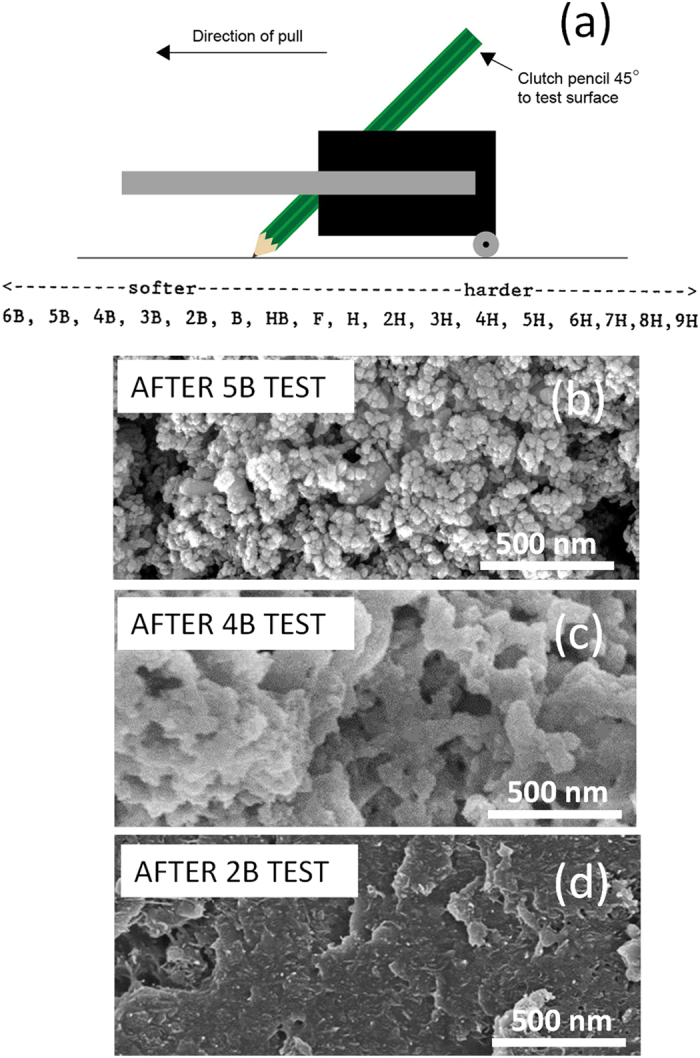
Pencil scratch tests. (**a**) Schematic representation of pencil scratch test along with the softness-hardness scale. (**b**) SEM image of the surface morphology of the TAPNC coating after 5B pencil harness test. (**c**) SEM image of the surface morphology of the TAPNC coating after 4B pencil harness test. (**d**) SEM image of the surface morphology of the TAPNC coating after 2B pencil harness test. Flattening of the surface features is clearly visible.

The mechanical durability of the present multilayer coatings was evaluated using dry pencil hardness test, dry and wet tape adhesion, and by Taber linear abraser. The target was to develop a coating with minimum 2B pencil hardness and a tape adhesion rating above 4B. According to ASTM D3363^43^ pencil hardness test method, a pencil with quantified hardness is dragged on the surface to be tested. Generally, the pencil is held in a carriage that is 45° and is pressed firmly on the surface while moving along it at a constant speed (Fig. 8a). The maximum pencil hardness that the surface can withstand before the pencil leaves a permanent mark is associated with its mechanical durability. The pencil hardness scale ranges from 10B (softest) to 10H (hardest). However, in general for testing the scale from 6B to 9H is used (Fig. 8a).

In Fig. 8b, SEM image of the surface is shown after testing the coating with a 5B pencil. No wear or scratch mark was visible and the multi-layer coatings remained superhydrophobic. A test using a 4B pencil (Fig. 8c) results in disruption of the initial surface morphology. However, the resultant surface features are sufficient to maintain a superhydrophobic state. Application of a 3B pencil on the multi-scale surface results in a similar morphology shown in Fig. 8c. However, the same experiment with a 2B pencil causes substantial removal of the nanoparticle layer and exposure of the underlying polymer coating (Fig. 8d). Hence, the multi-layer coatings can withstand up to 2B hardness while maintaining hydrophobicity with negligible contact angle hysteresis. In other words, zones damaged by the 2B pencil abrasion lost their superhydrophobic state (static water contact angles were reduced to 140–145°), but nonetheless, 10 μL droplets still slid off these damaged surfaces with negligible contact angle hysteresis at tilt angles close to 40°. Such kind of self-cleaning hydrophobic surfaces (slippery hydrophobic surfaces) exist in nature such as the skin of Nabib beetle (stenocara gracilipes) that recently inspired the design fog harvesting surfaces^44^,^45^.

**Figure 9:**
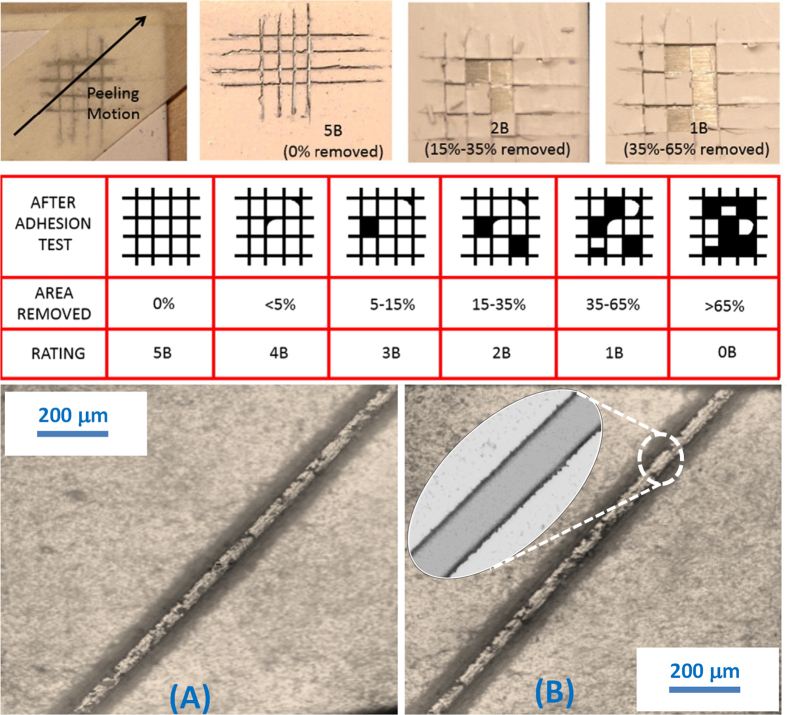
Top: Adhesion tests conducted on TAPNC coatings over aluminum substrates with their ratings according to the ASTM D3359. An adhesive tape is applied on the coating at a 45° angle relative to the cross-hatch cuts. After about one minute, the tape is peeled off and the sample is inspected. Middle: Indicative chart with damaged zone schematics, after the adhesion test, with their corresponding ratings according to the ASTM D3359. Bottom: (**A**) Optical microscope image of the scratch line before tape peel test. (**B**) Optical microscope image of the undamaged scratch line after tape peel test conforming to 2B Rating. The inset shows the undamaged edge details after tape peel.

Next, the tape peeling test was applied according to ASTM D3359 Method B^46^. Although the tape peel test was designed to test overall adhesion to the substrate for coatings, tape removal on superhydrophobic surfaces can also lead to partial destruction of the micro/nano-scale topography and thus the wetting properties are evaluated after each peeling cycle. In that sense, this test evaluates both adhesion to the substrate and cohesive adhesion. Tapes are classified according to the values of adhesion force to a reference substrate, reported as adhesion to steel in N/m. As this parameter (force/distance) increases, the tape peeling test becomes more destructive to the coating under investigation. In summary, the present coatings are first scratched with pencils of various hardness, then tested for dry tape peel adhesion and then soaked in water for one day and tape peel tests then repeated (see Fig. 9).

Approximately, 20% of the crosshatch patterns made by the 2B pencil were picked up by the tape peel action as seen in the top panel of Fig. 9. The rest of the crosshatch zone was undamaged as shown in the microscope images in Fig. 9 (bottom panel). Over the undamaged zones, no debris was detected after the tape peel action. Hence, in terms of adhesion strength to the metallic substrate (aluminum), the TAPNC coatings can be classified as rated 2B.

**Figure 10:**
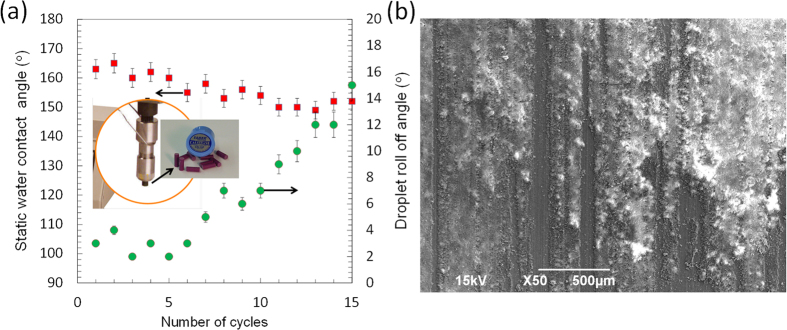
Changes in static water contact angle and water roll-off angles. (**a**) As the number of cycles approach 15, water droplet mobility on the surface declines, which is an indication of 17.5 kPa wear abrasion. The inset shows a photograph of the abrasion arm of the tester at the tip of which an abrading cylinder is mounted. (**b**) A representative SEM image showing appearance of wear marks at the end of 15 cycles under 17.5 kPa.

Next, we investigate the wear abrasion resistance of the TAPNC coatings under linear wear cycles. One of the most common durability testing methods for superhydrophobic coatings is cyclic wear abrasion tests^47^,^48^,^49^. The test is made by attaching a selected sandpaper grade at the bottom of a metallic weight (or vice versa) and by pulling this weight along the non-wetting surface (or vice versa) for a certain distance and repeat this in cycles. Wear abrasion resistance depends on the type of the abradant (sandpaper, cloth etc.) and the weight or applied pressure used for the testing. Figure 10 shows static water contact angle changes as well as water droplet roll-off angles as a function of number of linear wear cycles up to 15 cycles under 17.5 kPa load.

Although, a number of wear marks was observed at the end of the 15^th^ cycle on the surface, the scratched surface remained superhydrophobic. However, droplet roll-off angles increased close to 20°, indicating considerable decline in droplet mobility over the surface^50^. Compared to other reported works^47^,^48^,^49^,^51^, wear abrasion resistance of the TAPNC coatings will need to be improved further without forfeiting on the insect impact repellent properties. Further work will involve improving coating durability by designing a coating featuring a concept known as wear independent similarity performance^52^. Wear independent similarity requires that the coating retains its chemical properties and roughness geometry as outer surface material is removed under wear abrasion. The new exposed layers should have the required roughness as well as chemistry in order to be classified as coatings with wear independent similarity. They should also resist smoothing due to abrasion^52^.

**Figure 11:**
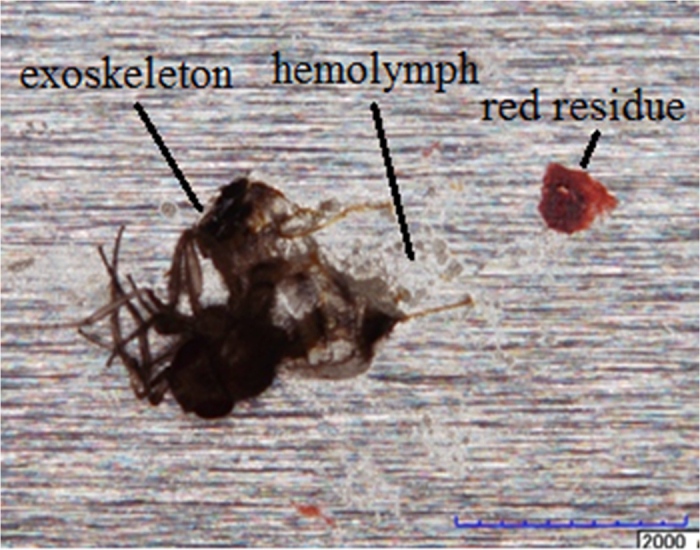
Photograph of a typical fruit fly insect residue on an aluminum foil surface.

**Figure 12:**
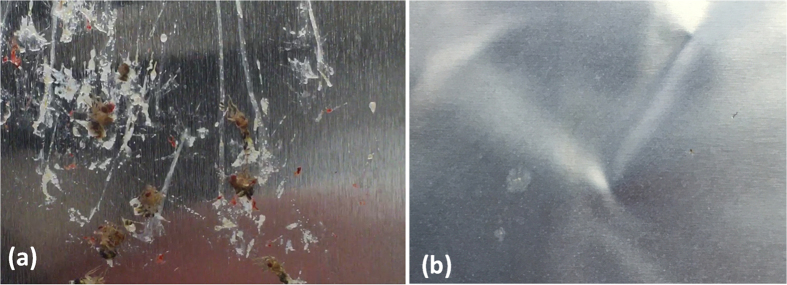
Photographs comparing the state of the surfaces after the wind tunnel experiments; (**a**) bare aluminum surface has many residues such as exoskeletons, hemolymph and red fluid stains whereas (**b**) the TAPNC coating only has a few hemolymph stains.

Three types of insect residues were observed from the wind tunnel testing. Those are identified as i) exoskeleton, ii) hemolymph and iii) red residue as shown Fig. 11 below. In Fig. 12, two photographs are presented comparing the resultant residue distribution on bare aluminum and the TAPNC coating. The photographs were taken after wind tunnel experiments in which 50 flightless fruit flies were released upstream and impacted the surfaces at 40–50 m/s. On average, of the 50 flies released per coupon, about 40 of these strike the airfoil cylindrical leading edge.

**Figure 13:**
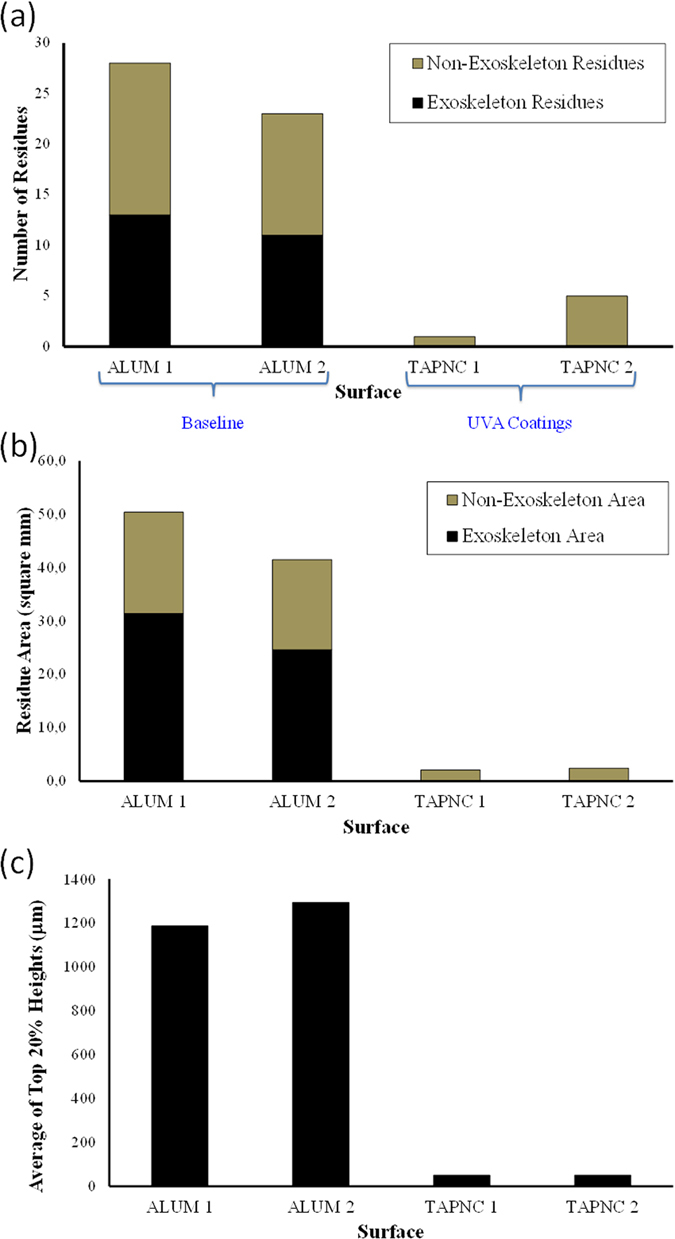
(**a**) Number of insect residues plotted versus aluminum (baseline) and TAPNC coating. (**b**) Residue area plotted versus aluminum (baseline) and TAPNC coating. (**c**) A plot showing the residues that make up of the top 20% highest roughness height versus aluminum (baseline) and TAPNC coating. Two separate experimental data are presented in which 50 flightless fruit flies were released upstream and impacted the surfaces at 40–50 m/s. On average, of the 50 flies released per coupon, about 40 of these strike the airfoil cylindrical leading edge.

As clearly seen in Fig. 12b, TAPNC coating features no exoskeleton residue compared to the baseline aluminum surface (Fig. 12a). Only a few hemolymph fluid stains can be seen on the superhydrophobic surface, TAPNC. A comparative quantitative residue analysis between the surfaces photographed in Fig. 12 is depicted in Fig. 13. The number of residues collected on both surfaces is plotted in Fig. 13a. As baseline two aluminum surfaces were tested and as superhydrophobic coatings, two separately fabricated TAPNC coatings were tested. As mentioned above, for each surfaces 50 insects were allowed to impinge on the surfaces at velocities close to 50 m/s.

On TAPNC surfaces only fluid residue in the form of non-exoskeleton is noticed. Moreover, the area associated with accumulated residue is drastically less on the TAPNC coatings as seen in Fig. 13b and is only made up of hemolymph with no exoskeleton. Furthermore, due to the contamination of the surfaces by insect residue, the surface roughness increases. This can be quantified and compared by calculating the top 20% residue height for both aluminum and TAPNC coatings as depicted in Fig. 13c.

The summary of data collected from Fig. 13 is shown in Table 1 which compares the performance of the coatings tested against a bare aluminum surface. The table shows the total number of residues collected on both surfaces. The total residue is made up of hemolymph, red residue and exoskeleton. As clearly seen, TAPNC has no exoskeleton attachment but some hemolymph fluid stains. The total area associated with residues and fluid stains are twenty times less on the TAPNC coating. The table also shows that for the residues that make up of the top 20% highest roughness height, the TAPNC coating has only 20 μm; whereas on aluminum surface this height exceeds 1 mm. This clearly indicates a two orders of magnitude reduction in the roughness caused by insect impact on the surfaces.

**Figure 14:**
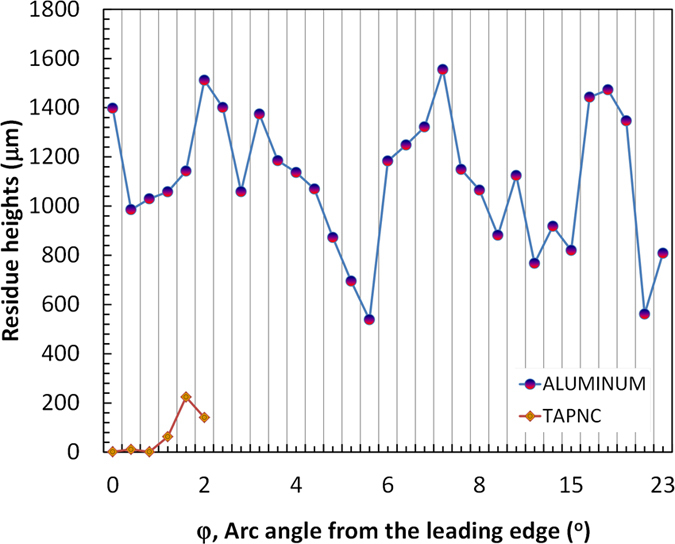
Insect residue heights as a function of Φ arc angle from stagnation line on leading edge radius. Red/blue circles represent residue accumulated on bare aluminum surface and orange/red diamonds represent residue on the TAPNC coating. The results indicate strong reduction in residue height and residue concentration (each symbol represent a single exoskeleton residue) for the TAPNC surface. The reductions are especially profound near the leading edge, where exoskeletons are most likely to occur in typical flight conditions.

The residue accumulation can be regarded as an additional undesired roughness, which may be measured as individual residue heights. Collectively, the resultant residue height can be quantified in several different ways such as the average residue height on each surface. However, since the amount of residue and number of insect impacts can vary from experiment to flight test, a scalable quantitative way was sought that would make the reported residue height more relevant to future flight tests where the number of insect impacts is not closely controlled. Figure 14 depicts residue heights (k) on two different surfaces, namely aluminum and TAPNC coating as a function arc angle (Φ) from stagnation line on leading edge radius (see Fig. 3). As can be seen in the figure, compared to the aluminum surface insect residue heights are much lower on the TAPNC surface, on average below 200 microns. Moreover, on the aluminum surface, for small arc angles (<5°) that are typically associated with the leading edge, there is a considerable amount of insect residue accumulation. In the case of TAPNC, the reductions are especially profound near the leading edge, where exoskeletons are most likely to occur in typical flight conditions. For arc angles exceeding 10°, insect residue on the TAPNC coating is also much less in terms of both heights and frequency.

## Conclusions

A new thermal alternating polymer nano-composite (TAPNC) coating has been developed that can repel high speed (50 m/s) insect impacts. The coatings also demonstrated 2B dry pencil hardness resistance and passed a scribe tape adhesion after a one day soak in water. Wear abrasion resistance was characterized as resilient under 17.5 kPa up to 15 linear abrasion cycles. Not all the superhydrophobic surfaces can repel insect residue under the wind tunnel experimental conditions studied herein. In fact, depending on their surface chemistry and surface texture, they can accumulate large amounts of insect residue on their surfaces. This superhydrophobic nanoparticle film however, is able to eliminate this residual accumulation due to the combination of its hydrophobicity and its relatively low average surface roughness which is not more than 500 nm. In order to give the coatings a pencil hardness of at least 2B, a minimum of six polymer-nanoparticle layer-by-layer coatings need to be applied. Melting of the polymer layer supporting the nanoparticle film is essential in order to mechanically attain the nanoparticles on the surface similar to a sand paper concept. While the performance in these simple lab durability tests is highly encouraging, advanced durability experiments specifically developed for aerospace coatings are needed in order to investigate the potential suitability of this thermal alternating polymer nanocomposite coating for realistic applications such as wind turbine blades or laminar flow airplane wings. Furthermore, the insect impact studies should be extended to flight conditions where a wide array of species and impact conditions can occur.

## Additional Information

**How to cite this article**: Bayer, I. S. *et al*. Thermal Alternating Polymer Nanocomposite (TAPNC) Coating Designed to Prevent Aerodynamic Insect Fouling. *Sci. Rep.* **6**, 38459; doi: 10.1038/srep38459 (2016).

**Publisher’s note:** Springer Nature remains neutral with regard to jurisdictional claims in published maps and institutional affiliations.

## Figures and Tables

**Table 1:** Insect impact residue statistics on aluminum compared to TAPNC surfaces.

Surface	Total number of residues	Number of exoskeletons	Residue area (mm^2^)	Exoskeleton area (mm^2^)	Average top 20% height (μm)
Aluminum	25.5	12	42	28	1240
TAPNC	3	0	2	0	20

## References

[b1] Nickel K. D. Washing installation for windshields or motor vehicles, aircraft, locomotives or the like. U.S. Patent No. 5,383,247 (1995).

[b2] Kok M., Smith J. G., Wohl C. J., Siochi E. J. & Young T. M. Critical considerations in the mitigation of insect residue contamination on aircraft surfaces–A review. Progress in Aerospace Sciences 75, 1–14 (2015).

[b3] Shankar P. N. Can insects seriously affect the power output of wind turbines? Current Science 81, 747–8 (2001).

[b4] Dalili N., Edrisy A. & Carrivaue R. A review of surface engineering issues critical to wind turbine performance, Renewable and Sustainable Energy Reviews 13, 428–438 (2009).

[b5] Joslin R. D. Aircraft Laminar Flow Control. Annual Review Fluid Mechanics 30, 1–29 (1998).

[b6] Freeman J. A. Studies in the Distribution of Insects by Aerial Currents - The Insect Population of the Air From Ground Level to 300 Feet, J. Anim. Ecol. 14, 128–154 (1945).

[b7] Coleman W. S. Roughness Due to Insects, Boundary Layer and Flow Control Volume 2, Lachmann G. V. ed. Pergamon Press, pp. 682–747 (1961).

[b8] Young T. M. & Humphreys B. Liquid Anti-contamination Systems for Hybrid Laminar Flow Control Aircraft-a Review of the Critical Issues and Important Experimental Results, Proceedings of the Institution of Mechanical Engineers, Part G: Journal of Aerospace Engineering 218, 267–277 (2004).

[b9] O’Donoghue D., Young T. M., Pembroke J. T. & O’Dwyer T. F. An investigation of surfactant and enzyme formulations for the alleviation of insect contamination on Hybrid Laminar Flow Control (HLFC) surfaces. Aerospace Science and Technology 6, 19–29 (2002).

[b10] Siochi E. . Engineered Surfaces for Mitigation of Insect Residue Adhesion. NASA Technical Paper NF1676L-15481 (2013).

[b11] Wohl C. J. . Evaluation of commercially available materials to mitigate insect residue adhesion on wing leading edge surfaces. Progress in Organic Coatings 76, 42–50 (2013).

[b12] Holmes B. J. & Obara C. J. Observation and implications of natural laminar flow on practical airplane surfaces. Journal of Aircraft 20, 993–1006 (1983).

[b13] Stenzel V., Wilke Y. & Hage W. Drag-reducing paints for the reduction of fuel consumption in aviation and shipping. Progress in Organic Coatings 70, 224–229 (2011).

[b14] Wicke K., Linke F., Gollnick V. & Kruse M. Insect Contamination Impact on Operational and Economic Effectiveness of Natural-Laminar-Flow Aircraft. Journal of Aircraft 53, 158–167 (2016).

[b15] Croom C. C. & Holmes B. J. Insect contamination protection for laminar flow surfaces. Langley Symposium on Aerodynamics Vol. 1, (ed. Stack S. H.) 539–555 (NASA, 1986).

[b16] Stenzel V. Method for Protecting the Surface of an Aircraft Against Contamination with Insect Residues and/or Icing, U.S. Patent No. 2012/0160963 A1 (2012).

[b17] Coleman W. S. Boundary Layer and Flow Control: It’s Principles and Applications (ed. Lachmann G. V.) Vol. II, 628–747 (Pergamon Press, 1961).

[b18] O’Donoghue D. . An investigation of surfactant and enzyme formulations for the alleviation of insect contamination on hybrid laminar flow control surfaces, Aerosp. Sci. Technol. 6, 19–29 (2002).

[b19] Kok M. & Young T. M. Evaluation of insect residue resistant coatings–Correlation of a screening method with a conventional assessment technique, Progress in Organic Coatings 77, pp. 1382–1390 (2014).

[b20] West C. J., Sutton D. A. & Bradley J. P. Aircraft component with aerodynamic surface Coating. U.S. Patent 20,100,242,996 (2010).

[b21] Lorenzi T. M., Wohl C. J., Penner R. K., Smith J. G. Jr. & Siochi E. J. Insect Residue Contamination on Wing Leading Edge Surfaces: A Materials Investigation for Mitigation *Proceedings of the 242nd American Chemical Society National Meeting and Exposition*, Denver, CO, US, 28 Aug.–1 Sep. (2011).

[b22] Milionis A., Krishnan K. G. & Loth E. Hemolymph drop impact outcomes on surfaces with varying wettability. Applied Surface Science 345, 36–43 (2015).

[b23] Kok M., Tobin E. F., Zikmund P., Raps D. & Young T. M. Laboratory testing of insect contamination with application to laminar flow technologies, Part I: Variables affecting insect impact dynamics. Aerospace Science and Technology 39, 605–613 (2014).

[b24] Larson C., Smith J. R. & Armstrong G. J. Current research on surface finishing and coatings for aerospace bodies and structures–a review. Transactions of the IMF 91, 120–132 (2013).

[b25] Kok M. & Young T. M. The evaluation of hierarchical structured superhydrophobic coatings for the alleviation of insect residue to aircraft laminar flow surfaces. Applied Surface Science 314, 1053–1062 (2014).

[b26] Yeong Y. H. . Temperature and humidity effects on superhydrophobicity of nanocomposite coatings. Applied Physics Letters 100(5), p.053112 (2012).

[b27] Milionis A., Bayer I. S. & Loth E. Recent advances in oil-repellent surfaces. International Materials Reviews 61(2), 101–126 (2016).

[b28] Wohl Jr, Christopher J. . Modified Surface Having Low Adhesion Properties To Mitigate Insect Residue Adhesion. U.S. Patent No. 20,150,251,217 (2015).

[b29] Wenzel R. N. Resistance of solid surfaces to wetting by water. Ind. Eng. Chem. 28, 988–994 (1936).

[b30] Cassie A. B. D. & Baxter S. Wettability of porous surfaces. Trans. Faraday Soc. 40, 546–551 (1944).

[b31] Doss J. R., Shanahan M. H., Wohl C. J. & Connell J. W. Synthesis, characterization and evaluation of urethane co-oligomers containing pendant fluoroalkyl ether groups. Progress in Organic Coatings 95, 72–78 (2016). 10.1016/j.porgcoat.2016.02.003 PMC7816764 33479554

[b32] Kok M., Mertens T., Raps D. & Young T. Influence of surface characteristics on insect residue adhesion to aircraft leading edge surfaces, Progress in Organic Coatings 76, 1567–1575 (2013).

[b33] Orchard M., Kohonen M. & Humphries S. The influence of surface energy on the self-cleaning of insect adhesive devices, The Journal of Experimental Biology 215, 279–286 (2011). 10.1242/jeb.063339 22189772

[b34] Scholz I. . Slippery surfaces of pitcher plants: Nepenthes wax crystals minimize insect attachment via microscopic surface roughness. The Journal of Experimental Biology 213, 1115–1125 (2010). 20228348 10.1242/jeb.035618

[b35] Howell D. & Behrends B. A review of surface roughness in antifouling coatings illustrating the importance of cutoff length. Biofouling 22, 401–410 (2006). 17178573 10.1080/08927010601035738

[b36] Scardino A. J., Zhang H., Cookson D. J., Lamb R. N. & Nys R. D. The role of nano-roughness in antifouling. Biofouling 25, 757–767 (2009). 20183134 10.1080/08927010903165936

[b37] Davis A., Mele E., Heredia-Guerrero J. A., Bayer I. S. & Athanassiou A. Omniphobic nanocomposite fiber mats with peel-away self-similarity. Journal of Materials Chemistry A 3, 23821–23828 (2015).

[b38] Milionis A., Dang K., Prato M., Loth E. & Bayer I. S. Liquid repellent nanocomposites obtained from one-step water-based spray. Journal of Materials Chemistry A 3, 12880–12889 (2015).

[b39] Steele A., Bayer I. & Loth E. Inherently superoleophobic nanocomposite coatings by spray atomization. Nano letters 9, 501–505 (2008). 10.1021/nl8037272 19099463

[b40] Zhu L. . Ice-phobic coatings based on silicon-oil-infused polydimethylsiloxane. ACS Applied Materials & Interfaces 5, 4053–4062 (2013). 23642087 10.1021/am400704z

[b41] Radaelli G. . Highly Effective Antiadhesive Coatings from pH-Modified Water-Dispersed Perfluorinated Acrylic Copolymers: The Case of Vulcanizing Rubber, Advanced Materials Interfaces 3(13), 1600069 (2016).

[b42] Steele A., Bayer I. & Loth E. Adhesion strength and superhydrophobicity of polyurethane/organoclay nanocomposite coatings. Journal of Applied Polymer Science 125 **(S1)**, E445–E452 (2012).

[b43] ASTM D3363-05e2, Standard Test Method for Film Hardness by Pencil Test, ASTM International, West Conshohocken, PA, www.astm.org (2011).

[b44] Wang L. & Zhou Q. Surface hydrophobicity of slippery zones in the pitchers of two Nepenthes species and a hybrid. Sci. Rep. 6, 19907 (2016). 26813707 10.1038/srep19907 PMC4728604

[b45] Mele E. . Zwitterionic Nanofibers of Super-Glue for Transparent and Biocompatible Multi-Purpose Coatings. Sci. Rep. 5, 14019 (2015). 26357936 10.1038/srep14019 PMC4566136

[b46] ASTM D3359-09e2, Standard Test Methods for Measuring Adhesion by Tape Test, ASTM International, West Conshohocken, PA, www.astm.org (2009).

[b47] Lu Y. . Robust self-cleaning surfaces that function when exposed to either air or oil. Science 347(6226), 1132–1135 (2015). 25745169 10.1126/science.aaa0946

[b48] Lu Y. . Creating superhydrophobic mild steel surfaces for water proofing and oil–water separation. Journal of Materials Chemistry A 2(30), 11628–11634 (2014).

[b49] Lu Y. . Preparation of superhydrophobic titanium surfaces via electrochemical etching and fluorosilane modification. Applied Surface Science 263, 297–301 (2012).

[b50] Milionis A., Languasco J., Loth E. & Bayer I. S. Analysis of wear abrasion resistance of superhydrophobic acrylonitrile butadiene styrene rubber (ABS) nanocomposites. Chemical Engineering Journal 281, 730–738 (2015).

[b51] Milne A. J. B. & Amirfazli A. Drop Shedding by Shear Flow for Hydrophilic to Superhydrophobic Surfaces. Langmuir 25, 14155–14164 (2009). 19685896 10.1021/la901737y

[b52] Steele A., Davis A., Kim J., Loth E. & Bayer I. S. Wear Independent Similarity. ACS Applied Materials & Interfaces 7, 12695–12701 (2015). 26018058 10.1021/acsami.5b00725

